# Development of novel small molecule EPHB3 inhibitors to treat neurodegenerative disease by targeting astrocyte‐mediated disease mechanisms

**DOI:** 10.1002/alz70859_104020

**Published:** 2025-12-25

**Authors:** Evan P Lebois, Chinnappa D Kodira, Robert D Hubbard, Darby R Schmidt

**Affiliations:** ^1^ Violet Therapeutics, Somerville, MA USA; ^2^ Mass General Brigham Ventures, Somerville, MA USA

## Abstract

**Background:**

Brain glial cells have emerged as key pathogenic drivers of Alzheimer’s disease (AD). We previously identified EPHB3 as a novel target that mediates cellular interactions between astrocytes and microglia in vivo to produce neuroinflammation and have now developed small molecule EPHB3 inhibitors with drug‐like properties. We dosed these novel inhibitors in vivo to test our therapeutic hypothesis that EPHB3 inhibition will attenuate reactive astrocyte gene signatures associated with AD neuropathology, thereby decreasing neuroinflammation and rescuing associated behavioral deficits in mouse models of amyloidosis (5xFAD) and tauopathy (PS19).

**Method:**

We synthesized a collection of small molecule EPHB3 inhibitors and characterized them using both an in vitro kinase assay and also a cellular NanoBRET assay, both employing human EPHB3. We subsequently demonstrated PK/PD of select compounds in an acute LPS model with ICV dosing. In vivo efficacy of the top EPHB3 inhibitor that emerged from these efforts, VT‐001, was further established in 14‐day EAE, 2‐month 5xFAD, and 3‐month PS19 chronic dosing studies. Unbiased single‐cell and single‐nucleus RNA‐seq (scRNA and snRNA‐seq), proteomics, and immunofluorescence were used to assess VT‐001 efficacy on astrocyte and microglial mechanisms in total spinal cord (EAE), as well as neocortex and hippocampus (5xFAD). Relevant behavioral outcomes were monitored by clinical scoring (EAE), elevated plus maze (5xFAD, PS19), and Morris water maze (5xFAD, PS19).

**Result:**

Our EPHB3 inhibitor VT‐001 significantly rescued behavioral deficits in EAE, 5xFAD, and PS19 models, as well as attenuated neuroinflammation evoked by ICV LPS in mice. The rescue of EAE and 5xFAD behavioral deficits was associated with a robust attenuation in reactive astrocyte signature genes, neuroinflammation genes, as well as Aβ plaque‐induced gene sets in astrocytes and microglia. Additionally, VT‐001 was found to be well‐tolerated with excellent CNS exposure across all in vivo studies. Finally, a next generation molecule, VT‐002 with improved potency was identified and characterized in LPS and rat tolerability studies.

**Conclusion:**

VT‐001 emerged as a potent and selective small molecule EPHB3 inhibitor that displays striking in vivo efficacy in neuroinflammation and neurodegeneration disease models. EPHB3 inhibitors in general and VT‐001 specifically, represent a highly promising opportunity for novel neurodegenerative disease therapeutics targeting astrocyte‐mediated disease mechanisms.

